# A new *Xilithus* species from Hubei, China (Araneae, Phrurolithidae)

**DOI:** 10.3897/BDJ.12.e130526

**Published:** 2024-08-20

**Authors:** Rongxin Liu, Jie Liu, Changyong Liu, Kuai Chen, Changhao Hu

**Affiliations:** 1 Qizimeishan National Nature Reserve Administration, Enshi, China Qizimeishan National Nature Reserve Administration Enshi China; 2 Hubei Key Laboratory of Regional Development and Environmental Response, Faculty of Resources and Environmental Science, Hubei University, Wuhan, China Hubei Key Laboratory of Regional Development and Environmental Response, Faculty of Resources and Environmental Science, Hubei University Wuhan China; 3 The State Key Laboratory of Biocatalysis and Enzyme Engineering of China, College of Life Science, Hubei University, Wuhan, China The State Key Laboratory of Biocatalysis and Enzyme Engineering of China, College of Life Science, Hubei University Wuhan China; 4 Hubei Broad Nature Technology Service Co., Ltd., Wuhan, China Hubei Broad Nature Technology Service Co., Ltd. Wuhan China

**Keywords:** guardstone spider, taxonomy, biodiversity, morphology

## Abstract

**Background:**

The genus *Xilithus* Liu & Li, 2023 contains 22 species, three of which are known from Hubei Province, China: *X.acerosus* (Yao, Irfan & Peng, 2019), *X.auritus* (Fu, Zhang & Zhang, 2016) and *X.xingdoushanensis* (Yao, Irfan & Peng, 2019).

**New information:**

One new *Xilithus* species from Hubei, China is described: *X.qizimeishanensis* Liu & Hu **sp. nov.** Morphological description, digital photos and distribution map are provided.

## Introduction

The spider family Phrurolithidae Banks, 1892 contains 25 extant genera and 404 species (World Spider Catalog 2024). *Xilithus* Liu & Li, 2023 is the seventh largest genus in the family and contains 22 species; four of them are known only from males and two from females (Liu et al. 2022, Kamura 2022, Lin et al. 2023, Mu and Zhang 2023, World Spider Catalog 2024). Species of *Xilithus* are usually small to medium spiders (2.5–5.0 mm), which have unique copulatory organ characters: males have a thin and long embolus, and a prolateral tegular apophysis; females have a pair of large atriums on epigynal plate (Liu et al. 2022, Mu and Zhang 2023). Eighteen *Xilithus* species have been found in China so far, three of which are known from Central China (Fu et al. 2016, Yao et al. 2019). Based on the specimen collected in Qizimeishan National Nature Reserve, Hubei, China, one new *Xilithus* species is described in the current paper.

## Materials and methods

The specimen examined in this study was preserved in ethanol absolute and deposited in the Centre for Behavioural Ecology and Evolution, College of Life Sciences, Hubei University in Wuhan. The specimen was examined using an OLYMPUS SZX7 stereo microscope. Photographs were taken with a LEICA M205 C stereo microscope, and final multifocal images were produced with Helicon Focus (Version 7.7.0). The epigyne was removed and treated in a warmed 0.1 mg/ml Protease K solution before study. The leg measurements are shown as total length (femur, patella, tibia, metatarsus, tarsus). All measurements in the text are given in millimetres. The map was created with ArcGis (Version 10.8.1).

The terminologies followed Liu et al. (2022). Abbreviations: **A** - atrium, **ALE** - anterior lateral eye, **AME** - anterior median eye, **B** - bursa, **CD** - copulatory duct, **CO** - opulatory opening, **CT** - connecting tube, **FD** - ertilization duct, **GA** - glandular appendage, **MS** - median septum, **PA** - posterior margin of atrium, **PLE** - posterior lateral eye, **PME** - posterior median eye, **S** - spermathecae. Spination: **d** - dorsal, **pl** - prolateral, **pv** - prolateral ventral, **rv** - retrolateral ventral.

## Taxon treatments

### Xilithus qizimeishanensis

Liu & Hu sp. nov.

5EB80EE0-B577-5807-900E-1204E226B747

8D8F0988-22E2-4315-97F9-43AD18E2911F

#### Materials

- **Type status:** Holotype. **Occurrence:** recordedBy: Changhao Hu (CBEE) & Mian Wei (CBEE); individualCount: 1; sex: female; lifeStage: adult; **Taxon:** kingdom: Animalia; phylum: Arthropoda; class: Arachnida; order: Araneae; family: Phrurolithidae; genus: Xilithus; **Location:** continent: Asia; country: China; countryCode: China/CN; stateProvince: Hubei; county: Xuanen; verbatimLocality: Qizimeishan National Nature Reserve, Shadaogou Town, Xueluozhai Forest Farm; verbatimElevation: 1338; verbatimLatitude: 29.74721°N; verbatimLongitude: 109.74536°E; **Event:** year: 2023; month: 7; day: 19; **Record Level:** institutionID: QZMS02704; institutionCode: CBEE

#### Description

**Female (Holotype)**: Total length 3.87, carapace 1.61 long, 1.43 wide; abdomen 2.11 long, 1.92 wide. Eye sizes and interdistances: AME 0.08, ALE 0.10, PME 0.07, PLE 0.09; AME–AME 0.06, AME–ALE 0.03, ALE–ALE 0.26, PME–PME 0.09, PME–PLE 0.06, PLE–PLE 0.36, AME–PME 0.08, ALE–PLE 0.08. Eye area 0.44 width, cephalic region 0.83 width. Median ocular area 0.23 long, anterior width 0.19, posterior width 0.20. Clypeal height 0.13. Labium 0.27 long, 0.19 wide. Sternum 0.99 long, 0.91 wide. Leg measurements: Ⅰ 5.74 (1.59, 0.41, 1.71, 1.32, 0.71), II 4.65 (1.29, 0.36, 1.27, 1.00, 0.73), III 4.26 (1.17, 0.41, 0.87, 1.08, 0.73), IV 6.47 (1.75, 0.46, 1.50, 1.72, 1.04). Leg formula: IV-I-II-III. Spination: femora I pl 3, femora II d 1 pl 2, femora III d 1, tibia I pv 7 rv 8, tibia II pv 8 rv 8, metatarsus I pv 4 rv 4, metatarsus II pv 5 rv 3.

**Figure 1.**
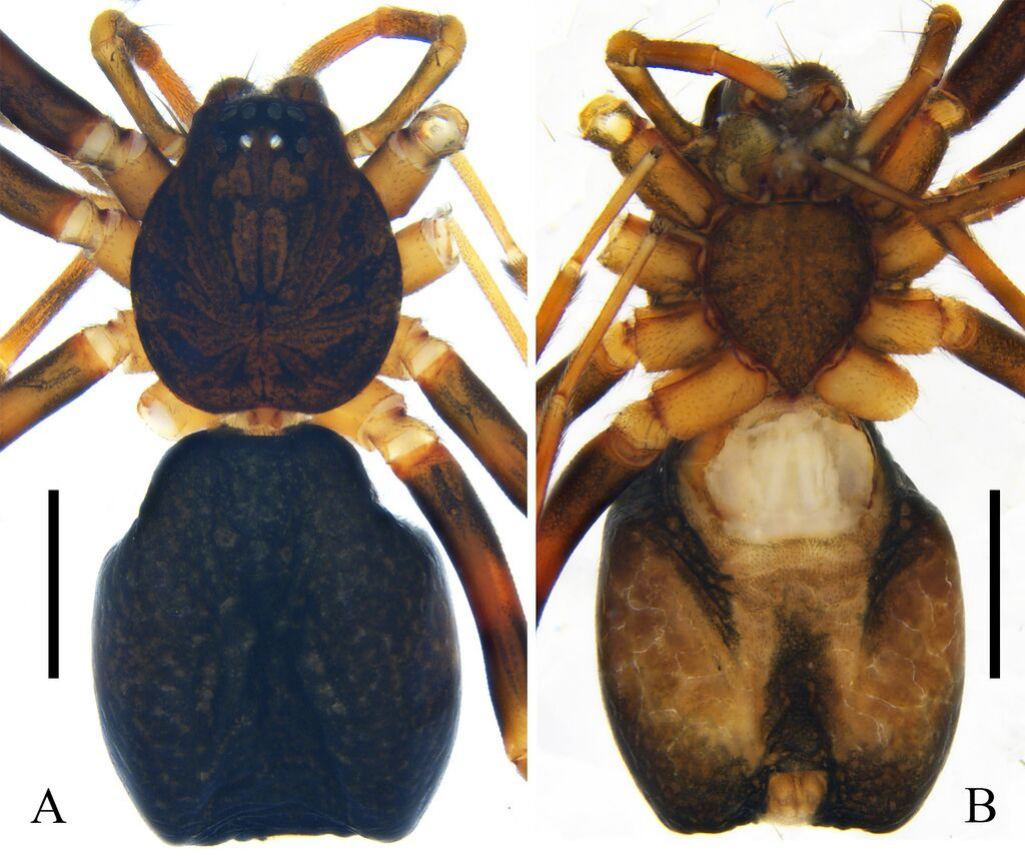
Habitus of female *Xilithusqizimeishanensis* Liu & Hu **sp. nov. A** dorsal view; **B** ventral view. Scale bars: 1 mm.

Colouration: Carapace reddish brown, radial furrows black. Labium and sternum brown. Legs yellow to brown. Abdomen: dorsum black; venter orange, posterior-lateral epigyne and anterior spinnerets black; spinnerets orange (Fig. 1A, B).

**Figure 2.**
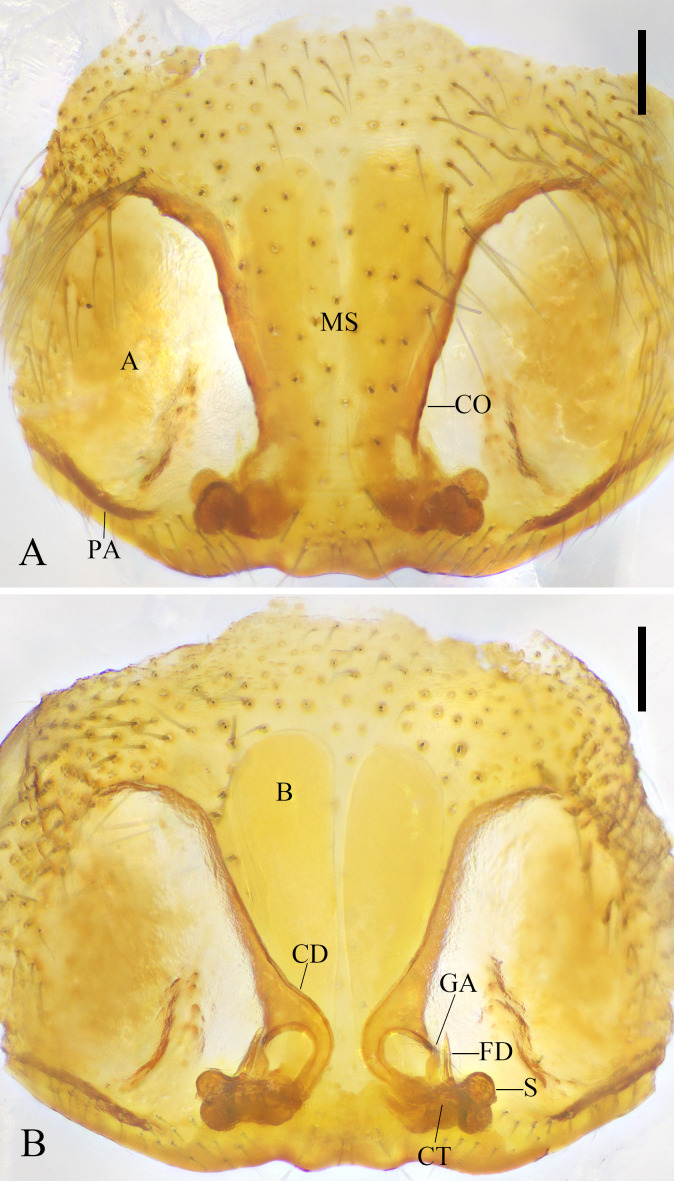
*Xilithusqizimeishanensis* Liu & Hu **sp. nov. A** Epigyne, ventral view; **B** Vulva, dorsal view. Scale bars: 0.1 mm.

Epigyne with pair of large oval atria (A). Median septum (MS) wide and trapezoidal. Copulatory openings (CO) trumpet-like, located at posterior margin of median septum (MS). Copulatory ducts (CD) short and C-shaped, the length of copulatory ducts (CD) almost twice the diameter of the spermathecae (S). Bursae (B) three times longer than wide, and exceed the anterior edge of the atria (A). Glandular appendages (GA) short, pointed anteriorly. Connecting tubes (CT) very short, between the base of glandular appendages (GA) and spermathecae (S). Spermathecae (S) double globular. Fertilization ducts (FD) short, located interiorly on spermathecae (S), pointed anteriorly (Fig. 2A, B).

**Male**: unknown.

#### Diagnosis

The female of the new species resembles that of *X.pseudostella* (Fu, Jin & Zhang, 2014) (Fu et al. 2014) in having a wide and trapezoidal median septum (MS) and two double globular spermathecae (S), but can be recognized by: 1. copulatory openings (CO) located posteriorly (vs. anteriorly); 2. short and curved copulatory ducts (CD) (vs. long and straight); 3. length of copulatory ducts (CD) almost twice the diameter of the spermathecae (S) (vs. three times); 4. length of bursae (B) almost equal to the diameter of the atria (A) (vs. shorter than the diameter of the atria (A)); 5. glandular appendages (GA) located posteriorly (vs. medially); 6. spermathecae (S) located laterally on connecting tubes (CT) (vs. posteriorly).

#### Etymology

The specific name is derived from the type locality.

#### Distribution

**Figure 3.**
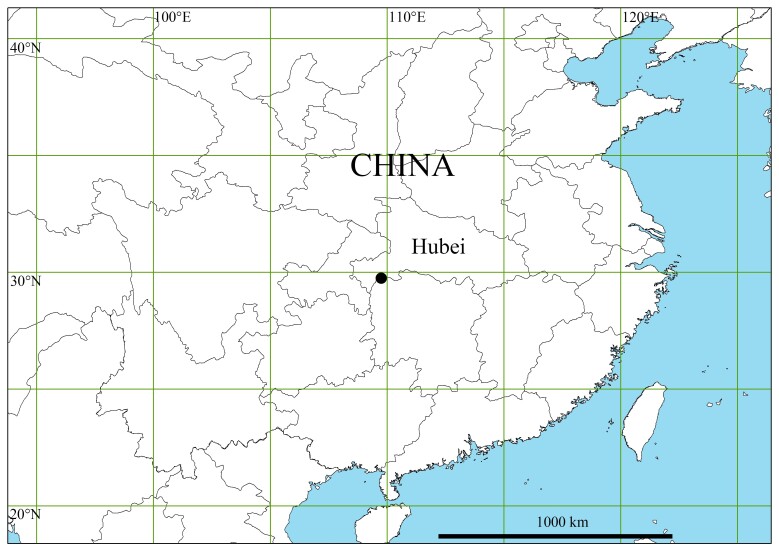
Collection locality of *Xilithusqizimeishanensis* Liu & Hu **sp. nov.** in Hubei, China.

Known only from the type locality (Fig. 3).

#### Notes

Liu et al. (2022) divided *Xilithus* species into five species groups: *acerosus*-, *acutus*-, *leibo*-, *lingyun*-, and *ovatus*-groups. The two large oval atria (A) separated by nose-shaped median septum (MS) and the organs of endogyne gathered together on ental of median septum (MS) of *X.qizimeishanensis* Liu & Hu **sp. nov.** show that the new species clearly belongs to *lingyun*-group.

## Supplementary Material

XML Treatment for Xilithus qizimeishanensis
